# Auditory pathway abnormalities in Parkinson's disease

**DOI:** 10.1055/s-0045-1801844

**Published:** 2025-02-06

**Authors:** Rafaela Valiengo de Souza, Liliane Aparecida Fagundes Silva, Carla Gentile Matas

**Affiliations:** 1 Universidade de São Paulo, Faculdade de Medicina, Departamento de Fisioterapia, Fonoaudiologia e Terapia Ocupacional, São Paulo SP, Brazil.

**Keywords:** Auditory Cortex, Parkinson Disease, Hearing Loss, Evoked Potentials, Auditory, Event-Related Potentials, P300

## Abstract

**Background** Parkinson's disease (PD) is a degenerative, progressive, chronic disease that mainly affects the central nervous system, caused by dopamine deficiency. One of the ways to evaluate the central nervous system is with auditory evoked potentials (AEP).

**Objective** To characterize the audiometric responses, and the auditory brainstem response (ABR), and cortical auditory evoked potentials (CAEP) in individuals with PD.

**Methods** Thirty-two patients aged between 40 and 81 of both sexes were assessed, 16 with PD (study group [SG]) and 16 without PD (control group [CG]) matched for sex and age. The subjects were assessed using pure tone audiometry, ABR with click stimuli, and CAEP using the oddball paradigm with tone burst and speech stimuli. The results were compared between the groups using a repeated measures analysis of variance (ANOVA) test.

**Results** In pure-tone audiometry, significantly higher hearing thresholds were found in the SG at 6 and 8 kHz. For the ABR, no differences were observed between groups. The CAEP analysis did not find statistical differences in the latencies between the groups, however, the SG presented smaller amplitudes of P1-N1, P2-N2, and N2-P3 than the CG.

**Conclusion** The results of this study showed a significantly higher threshold in higher frequencies in PD. Although no differences were observed at the brainstem level, the decrease in amplitude of all components in patients with PD in the CAEP suggests a deficit in both automatic and attentional cortical processing of acoustic stimuli.

## INTRODUCTION

Increased life expectancy is a preponderant factor in epidemiological transition, requiring greater attention to aging-related chronic and degenerative diseases, 1 including Parkinson's disease (PD), 2 3 whose predominance is 1 to 2 per 1000 people in the general population and 1 per 100 in those over than 65 years old.

PD was described by James Parkinson in 1917 as a paralysis agitans, 4 and its classic clinical condition is characterized by motor signs such as tremors, stiffness, bradykinesia, and postural instability, due to the degeneration of nigrostriatal dopaminergic neurons at the brain nuclei. 5 PD is currently recognized as a disease with a broader and more diffuse spectrum involving not only the dopaminergic neurodegeneration of the motor system but also the impairment of various organs and systems manifested with nonmotor signs and symptoms. 6 7 8

Although clinical manifestations are the basis for diagnosis and analysis of the progression of the disease, the process of neuropathological degeneration can precede the appearance of these signs and symptoms by several years, which become evident with extensive dopaminergic neuronal loss in the substantia nigra in more advanced degenerative conditions. 9

Complaints related to reduced hearing sensitivity and, consequently, the search for an audiological diagnosis in individuals with the disease are often neglected by patients and professionals, to the detriment of the significant motor, psychiatric, cognitive, and autonomic impairments present in the disease. 10 However, hearing loss is common in the elderly and contributes to social isolation, reduced independence, and functional capacities, as well as worsening cognitive impairments and emotional compromises. 11 Thus, the effects of hypoacusis can be minimized when proper diagnosis and auditory rehabilitation strategies are performed, improving the quality of life of this section of the population.

Studies have found that the prevalence of hearing loss exceeds 70% in individuals with PD and that this incidence is higher than that observed in elderly people without the disease. 12 13 14 15

In addition to alterations in the peripheral auditory system, leading to hearing loss in this population, it is known that alterations in temporal perception are also attributed to the cognitive deficits in attention and memory that can be observed in PD, maintaining a close relationship with impairment in noradrenergic and cholinergic neurotransmitters. 16 17 Therefore, alterations in the Central Auditory Nervous System (CANS) should also be investigated.

The central hearing has been assessed with electrophysiological tests, such as auditory evoked potentials (AEP). They assess the neuroelectric activity in the auditory pathway, from the auditory nerve to the brain cortex, as a response to an acoustic stimulus or event. AEPs can be recorded with surface electrodes placed in various regions of the scalp. They are generated by the sequential and synchronic activation of nervous fibers throughout the auditory pathway. 18

AEPs have been studied to assess hearing in various situations, including degenerative neurological diseases.

In a systematic review of AEP in PD, it was observed that the presence of processes that compromise the neuroelectrophysiological conduction of the afferent auditory pathways is controversial in individuals with PD. It has not been possible to date to establish a pattern that clearly defines whether there are and what types of AEP abnormalities are present in this population. 19

Therefore, the evaluation of the peripheral and central auditory pathways is important including auditory brainstem response (ABR) and cortical auditory evoked potential (CAEP) assessment. Electrophysiological evaluation could support the identification of whether the functional integrity of the peripheral and central auditory pathways in patients with PD and monitoring of the progression of the disease, as well as the benefit of the treatment performed.

The hypothesis of this study is that individuals with PD would have delayed latency of both ABR and CAEP responses, and reduced amplitude of CAEP components.

Considering this, the present study aimed to characterize the audiometric responses, and the ABR and CAEP in individuals with PD.

## METHODS

A prospective cross-sectional study was performed with adults and elderly people diagnosed with PD by a neurologist. This study was realized at the Department of Audiology and Speech Therapy, Physiotherapy and Occupational Therapy, University of Sao Paulo Medical School, São Paulo, Brazil. The research was approved by the institutional Ethics Committee, under research protocol no. 3.241.829.

The exclusion criteria were as follows: consuming alcohol or other illegal drugs; occupational exposure to high sound pressure levels; excess cerumen; history or presence of middle ear alterations; presence of Type B or C tympanometric curve 20 ; presence of moderately severe, severe, or profound sensorineural hearing loss. 21

The study comprised 32 individuals, over 40 years old. The study group (SG) was composed of 16 individuals with PD, and the control group (CG) was composed of 16 individuals without PD sex and age-matched to SG. PD patients were recruited at a specific Parkinson's Association, and individuals without PD were recruited from a convenience sample.

Initially, an anamnesis, otoscopy, and acoustic Immittance measurements were performed to verify the patient's eligibility according to the exclusion criteria.

After this, pure tone audiometry (PTA) was performed, using an audiometer manufactured by Grason Standler, model GSI 61, in a soundproof booth, at frequencies from 0.25 to 8 kHz by air conduction. When any frequency between 0.5 and 4 kHz had a threshold above 20 dB HL, the bone conduction threshold was investigated.

The electrophysiological assessment lasted ∼60 minutes. During the recording of this procedure, the subject was seated comfortably in a recliner in an acoustically and electrically treated room.

To assess AEPs, the intelligent hearing system equipment, model Smart EP, with insert earphones, model ER 3A was used. The individual's skin was first cleaned with abrasive paste, and the Ag/AgCl electrodes were fixed on the scalp with electrolytic paste and micropore tape in specific positions, according to International Electrode System IES 10–10, as follows: the ground electrode was placed on the forehead, the reference electrodes were placed on the left (M1) and right mastoids (M2), and the active electrode was placed on the vertex (Cz) to record CAEP and on the forehead (Fz) to record ABR.

For the ABR, a rarefied polarity click stimulus at 80 dBnHL was used, presented monaurally at a presentation rate of 27.7 stimuli per second, totaling 2000 stimuli. Two recordings were obtained to check their reproducibility and confirm the presence of a response.

The CAEP was recorded using an oddball paradigm. Acoustic speech stimuli (syllables /ba/ and /da/) and tone burst stimuli (at 1000 and 2000 Hz) were presented monaurally at 75 dBnHL, at a presentation rate of 1.1 stimuli per second, totaling 300 stimuli, of which 15 to 20% were target ones. The standard speech stimuli were the syllable /ba/ and the target ones were the syllable /da/. The standard tone burst stimuli were at 1000 Hz, and the target ones were at 2000 Hz. Subjects were instructed to pay attention to the target stimuli and count how many times the target event occurred.

For the ABR were analyzed the absolute latencies of waves I, III, and V and the interpeaks I-III, III-V, and I-V in milliseconds (ms). For the CAEP investigation, the P1, N1, P2, and N2 components were identified and analyzed regarding their latency and amplitude peak-to-peak in the trial corresponding to the standard stimuli, while P3 was identified and analyzed in the trial corresponding to the target stimuli.

To analyze the data, the unpaired *t* -test was used to compare the ages between the two groups. To compare the results of PTA and ABR between the groups, we used the mixed ANOVA test of repeated measures, in which the ear was considered as a factor of repeated measure, and the group was considered as a factor between subjects. Finally, to compare the CAEP results between the groups, a mixed repeated-measures ANOVA was used, in which ear and stimulus were considered as repeated-measures factors, and the group was considered as a between-subjects factor. Tukey's test was used for the post hoc analysis.

For the variables that showed a significant difference between the groups, a correlation analysis was performed with age, time of diagnosis, and time of drug use using Spearman's Rho test or Pearson test.

A significance level of 5% (α = 0.05) was adopted for all analyses.

## RESULTS

SG comprised 16 individuals (eight females and eight males), aged 40 to 81 years (58.13 ± 11.03), and CG had another 16 individuals, matched for sex and age with SG individuals, aged 42 to 81 years (57.75 ± 10.66), with no statistically significant difference between the groups for the age factor ( *t* = −0.098; *p* = 0.923).

SG individuals had been diagnosed between 0.6 to 21.5 years ago (7.22 ± 6.40) and had been undergoing treatment for 0.5 to 21.3 years (7.12 ± 6.39), all of whom were taking Prolopa ( Table 1 ).

**Table 1:** Characterization of study group individuals

Subject	Time since diagnosis (years)	Time of treatment (years)	Other comorbidities	Other drugs they take
1	2.5	2.4	Denied	Denied
2	19.8	19.6	Hypertension /diabetes	Losartan 25 mg (for 5 years) Metformin (for 6 years)
3	21.5	21.3	Hypertension	Losartan 50 mg and Hydrochlorothiazide 25 mg (for 15 years)
4	15.2	15.2	Hypertension /diabetes	Chlortalidone, Amlodipine, Somalgin, Nesina, and Metformin (for 2 years)
5	8.4	8.3	Denied	Denied
6	7.4	7.4	Denied	Denied
7	6.8	6.6	Denied	Denied
8	2.4	2.3	Denied	Denied
9	5.6	5.4	Denied	Denied
10	2.3	2.3	Denied	Denied
11	10.1	10.0	Denied	Denied
12	3.0	2.8	Denied	Denied
13	3.9	3.6	Hypertension	Simvastatin and Losartan 50 mg (for 3 years)
14	4.0	3.9	Diabetes	Forxiga (for 8 years)
15	0.6	0.5	Hypertension	Atorvastatin 80 mg and Atenolol 25 mg (for 7 years)
16	2.2	2.0	Denied	Denied

**Figure 1:**
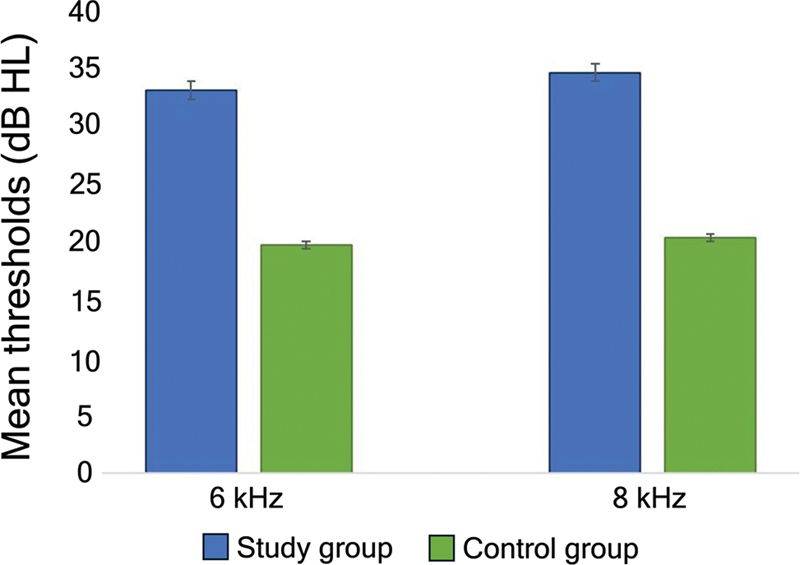
Comparison of the mean hearing thresholds at 6 and 8 kHz between the two groups.

**Figure 2:**
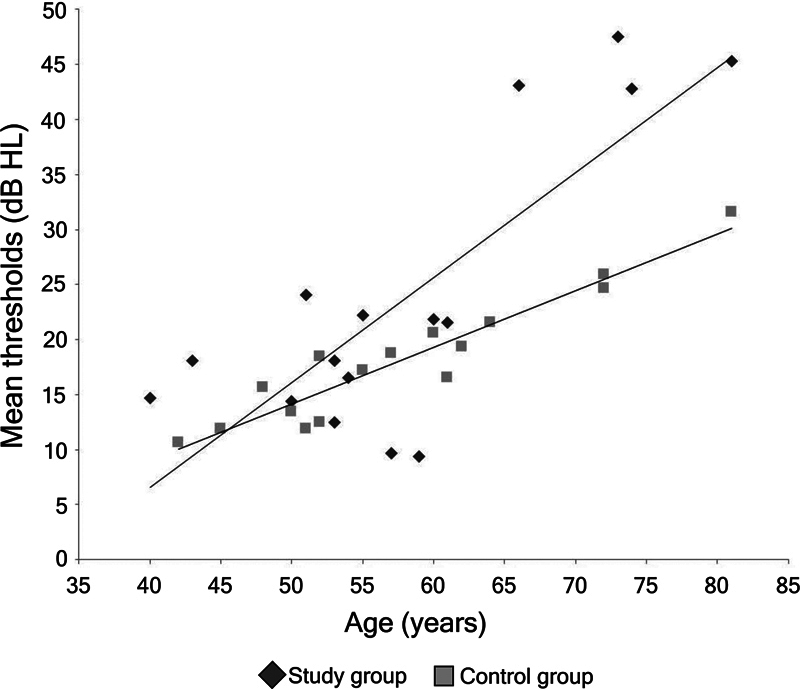
Scatter plot between age and mean hearing thresholds for each group.

The hearing loss was more prevalent in the SG ( *p* = 0.049), with 12 patients (75%) in the SG having hearing loss, while in the CG hearing loss was observed in only six patients (37.5%). The descriptive analyses of the PTA are shown in Table 2 . The PTA at 1 kHz showed in the left ear higher hearing thresholds than in the right ear ( *F* = 9.01; *p* = 0.005). In addition, significantly higher thresholds were found in SG only at 6 kHz ( *F* = 4.76; *p* = 0.037) and 8 kHz ( *F* = 4.52; *p* = 0.042), regardless of the ear ( Figure 1 ). The mean thresholds (between the frequencies of 0.25 and 8 kHz in both ears) had a positive correlation with age, and older patients had higher hearing thresholds ( *r* = 0.765; *p* < 0.001) ( Figure 2 ).

**Table 2:** Descriptive analysis of the hearing thresholds obtained in the ATL for each frequency in decibel hearing level (dB HL)

	Group	Ear	Median	Mean	SD	Minimum	Maximum
**0.25 kHz**	SG	RE	17.50	16.25	8.06	0.00	25.00
		LE	17.50	20.00	10.65	5.00	45.00
	CG	RE	12.50	14.38	7.04	5.00	25.00
		LE	15.00	14.37	6.55	5.00	25.00
**0.5 kHz**	SG	RE	17.50	15.00	8.17	0.00	25.00
		LE	17.50	18.13	12.09	5.00	50.00
	CG	RE	15.00	15.63	5.12	5.00	25.00
		LE	15.00	15.63	6.55	5.00	25.00
**1 kHz**	SG	RE	15.00	14.38	7.27	0.00	25.00
		LE	15.00	18.44	11.79	5.00	55.00
	CG	RE	15.00	15.63	5.12	5.00	25.00
		LE	20.00	18.44	6.25	5.00	25.00
**2 kHz**	SG	RE	20.00	17.81	8.94	5.00	40.00
		LE	17.50	21.56	13.75	10.00	55.00
	CG	RE	17.50	18.44	6.76	10.00	30.00
		LE	17.50	18.44	7.69	10.00	35.00
**3 kHz**	SG	RE	20.00	22.81	17.51	5.00	70.00
		LE	20.00	27.50	16.33	10.00	65.00
	CG	RE	17.50	19.06	6.12	10.00	35.00
		LE	15.00	18.44	8.70	10.00	40.00
**4 kHz**	SG	RE	15.00	25.63	20.89	10.00	85.00
		LE	22.50	29.38	19.23	10.00	70.00
	CG	RE	17.50	20.00	10.33	10.00	45.00
		LE	20.00	22.19	10.80	10.00	50.00
**6 kHz**	SG	RE	25.00	31.56	24.13	10.00	100.00
		LE	25.00	34.38	24.69	15.00	95.00
	CG	RE	20.00	21.25	10.08	10.00	50.00
		LE	15.00	18.13	5.74	10.00	30.00
**8 kHz**	SG	RE	25.00	33.13	26.26	10.00	105.00
		LE	25.00	35.94	27.82	10.00	105.00
	CG	RE	20.00	20.63	5.74	10.00	30.00
		LE	20.00	20.00	5.77	10.00	35.00

Abbreviations: CG, control group; kHz, kilo Hertz; LE, left ear; RE, right ear; SD, standard deviation; SG, study group.

ABR analysis included a descriptive analysis of the absolute latencies of waves I, III, and V, and the latencies of interpeak intervals I-III, III-V, and I-V per group and ear ( Table 3 ). There were no statistically significant differences between the groups, however, longer absolute latency of the wave V was obtained in the left ear, regardless of the group ( *F* = 8.32; *p* = 0.007).

**Table 3:** Descriptive analysis of absolute latencies of waves I, III, and V, and latencies of interpeak intervals I-III, III-V, and I-V per group and ear in milliseconds (ms)

	Group	Ear	Median	Mean	SD	Minimum	Maximum
**Wave I**	SG	RE	1.59	1.63	0.14	1.40	1.83
		LE	1.65	1.68	0.16	1.50	1.98
	CG	RE	1.68	1.65	0.13	1.35	1.83
		LE	1.63	1.61	0.11	1.43	1.85
**Wave III**	SG	RE	3.69	3.71	0.19	3.45	4.15
		LE	3.75	3.76	0.12	3.56	3.98
	CG	RE	3.79	3.73	0.19	3.20	3.93
		LE	3.79	3.75	0.11	3.53	3.90
**Wave V**	SG	RE	5.62	5.58	0.21	5.30	6.10
		LE	5.73	5.67	0.18	5.35	5.95
	CG	RE	5.59	5.57	0.17	5.15	5.83
		LE	5.61	5.62	0.15	5.25	5.83
**Interpeak interval I-III**	SG	RE	2.09	2.14	0.21	1.79	2.53
		LE	2.14	2.11	0.16	1.78	2.38
	CG	RE	2.14	2.09	0.22	1.48	2.38
		LE	2.18	2.17	0.09	2.03	2.33
**Interpeak interval III-V**	SG	RE	1.84	1.85	0.14	1.63	2.10
		LE	1.91	1.91	0.15	1.55	2.13
	CG	RE	1.84	1.83	0.14	1.63	2.03
		LE	1.89	1.76	0.11	1.67	2.05
**Interpeak interval I-V**	SG	RE	4.01	4.01	0.22	3.73	4.50
		LE	4.07	4.03	0.28	3.45	4.52
	CG	RE	3.98	3.93	0.17	3.43	4.13
		LE	4.05	4.02	0.12	3.80	4.20

Abbreviations: CG, control group; LE, left ear; RE, right ear; SD, standard deviation; SG, study group.

The CAEP analysis included a descriptive analysis of P1, N1, P2, N2, and P3 latency values ( Table 4 ) and P1-N1, P2-N2, and N2-P3 amplitudes ( Table 5 ) with each acoustic stimulus.

**Table 4:** Descriptive analysis of LLAEP component latencies obtained with both stimuli in milliseconds (ms)

		Group	Ear	N	Median	Mean	SD	Minimum	Maximum
**Tone-burst**	**P1**	SG	RE	16	63.00	67.25	21.21	38.00	102.00
			LE	16	67.00	75.38	23.29	42.00	121.00
		CG	RE	16	54.00	58.86	16.01	38.00	93.00
			LE	16	56.00	58.69	18.57	39.00	115.00
	**N1**	SG	RE	16	104.50	104.00	19.87	70.00	135.00
			LE	16	100.00	101.50	19.08	62.00	133.00
		CG	RE	16	98.00	102.19	23.43	84.00	122.00
			LE	16	103.00	99.06	22.71	39.00	140.00
	**P2**	SG	RE	16	183.00	183.38	28.66	140.00	258.00
			LE	16	177.50	182.81	35.34	120.00	251.00
		CG	RE	16	185.00	181.56	31.33	140.00	255.00
			LE	16	172.50	173.13	20.64	146.00	212.00
	**N2**	SG	RE	16	223.00	227.75	34.62	187.00	323.00
			LE	16	201.00	213.94	41.43	148.00	289.00
		CG	RE	16	261.50	249.38	37.06	179.00	311.00
			LE	16	217.50	228.06	38.03	148.00	289.00
	**P3**	SG	RE	16	351.00	344.69	43.99	248.00	401.00
			LE	16	332.50	334.50	27.32	300.00	395.00
		CG	RE	16	346.00	349.13	40.93	288.00	416.00
			LE	16	317.00	326.00	37.77	245.00	373.00
**Speech**	**P1**	SG	RE	16	71.00	69.81	10.06	51.00	88.00
			LE	16	76.00	72.31	12.38	36.00	85.00
		CG	RE	16	72.00	70.25	16.49	43.00	100.00
			LE	16	70.00	70.31	18.47	36.00	120.00
	**N1**	SG	RE	16	109.00	105.50	15.47	77.00	130.00
			LE	16	110.50	111.31	17.59	66.00	143.00
		CG	RE	16	114.00	112.88	17.84	81.00	153.00
			LE	16	115.50	115.19	23.54	66.00	179.00
	**P2**	SG	RE	16	185.00	186.94	23.06	122.00	227.00
			LE	16	190.50	187.07	33.12	87.00	232.00
		CG	RE	16	78.50	188.19	35.81	121.00	269.00
			LE	16	189.00	189.88	42.12	87.00	260.00
	**N2**	SG	RE	16	232.50	241.63	44.11	167.00	351.00
			LE	16	229.00	231.69	56.03	107.00	341.00
		CG	RE	16	232.50	245.25	42.30	165.00	326.00
			LE	16	260.50	251.81	50.99	107.00	312.00
	**P3**	SG	RE	16	330.00	329.69	34.53	275.00	412.00
			LE	16	334.00	333.13	37.30	267.00	385.00
		CG	RE	16	343.50	346.93	45.05	272.00	456.00
			LE	16	323.50	321.31	29.06	281.00	386.00

Abbreviations: CG, control group; LE, left ear; N, sample number; RE, right ear; SD, standard deviation; SG, study group.

**Table 5:** Descriptive analysis of LLAEP component amplitudes obtained with both stimuli in microvolts (µV)

		Group	Ear	N	Median	Mean	SD	Minimum	Maximum
**Tone-burst**	**P1- N1**	SG	RE	16	3.52	3.74	2.39	0.07	8.14
			LE	16	3.78	3.77	2.05	0.65	8.15
		CG	RE	16	3.94	4.72	3.13	0.68	11.40
			LE	16	4.53	5.24	2.59	2.42	11.79
	**P2-N2**	SG	RE	16	2.49	2.60	1.24	0.80	4.93
			LE	16	1.89	2.24	1.17	0.95	4.47
		CG	RE	16	4.67	4.37	1.76	1.10	7.77
			LE	16	3.47	3.25	1.57	0.76	5.85
	**N2-P3**	SG	RE	16	4.23	5.02	4.59	0.62	19.17
			LE	16	3.08	4.73	3.52	1.01	11.18
		CG	RE	16	5.74	6.89	4.87	1.97	20.13
			LE	16	7.45	7.38	4.05	1.39	15.80
**Speech**	**P1- N1**	SG	RE	16	2.24	2.83	1.87	0.93	7.12
			LE	16	2.52	3.26	1.87	0.71	7.60
		CG	RE	16	5.08	5.35	2.87	0.88	9.62
			LE	16	4.38	5.54	3.24	1.87	11.31
	**P2-N2**	SG	RE	16	2.84	2.57	1.00	0.63	4.35
			LE	16	1.87	2.52	2.12	0.31	7.35
		CG	RE	16	2.67	2.84	1.00	1.60	5.49
			LE	16	2.50	2.82	2.02	0.42	8.07
	**N2-P3**	SG	RE	16	5.07	5.04	3.36	0.76	10.99
			LE	16	5.47	4.90	3.52	0.32	11.77
		CG	RE	16	8.11	8.62	6.00	0.73	22.40
			LE	16	6.48	8.09	6.41	0.43	21.90

Abbreviations: CG, control group; LE, left ear; N, sample number; RE, right ear; SD, standard deviation; SG, study group.

With regard to latency, there was no significant difference for the ear, stimulus, or group factor in either of the CAEP components ( *p* > 0.05).

**Figure 3:**
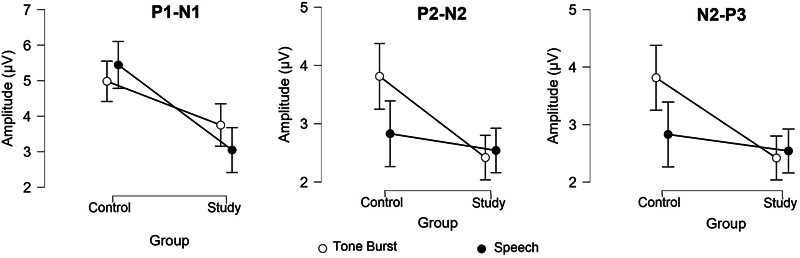
Comparison of P1-N1, P2-N2 and N2-P3 amplitudes between both groups.

Concerning amplitude measurements, an interaction effect between the group and stimulus was observed for the P1-N1 amplitude, with the SG showing reduced amplitude compared with the CG only for the speech stimulus ( *t* = 2.849; *p* = 0.034). For the P2-N2 amplitude, there was an interaction effect between the group and stimulus, with the SG having a reduced amplitude compared with the CG for the tone burst stimulus ( *t* = 2.910; *p* = 0.027). In addition, there was a significant difference according to stimulus only in the CG, where greater amplitudes were observed with the tone burst stimulus compared with the speech stimulus ( *t* = 3.084; *p* = 0.021). As for the N2-P3 amplitude, there were significant differences between the groups, with the SG having a lower amplitude compared with the CG ( Figure 3 ).

There was no significant correlation between the results of the P1-N1, P2-N2, and N2-P3 amplitudes and age, diagnosis time, and treatment time ( *p* > 0.05).

## DISCUSSION

This study aimed to characterize the audiometric responses and the ABR and CAEP with both tone burst and speech stimuli in adults and elderly people diagnosed with PD (SG) in comparison with ones without PD diagnosis (CG).

SG individuals had been diagnosed 0.6 to 21.5 years before and had begun treatment 0.5 to 21.3 years before; all of them were taking Prolopa, corroborating with other ones, which also verified that the drug most used by PD patients was Prolopa. 22 23

The PTA showed that individuals with PD had higher hearing thresholds than CG subjects at 6 and 8 kHz. This finding corroborates previous studies in which the authors also observed a worsening of hearing thresholds in PD patients, especially at higher frequencies, even without clinical symptoms. 10 12 13 24 25 26 27 28 Their hypothesis is that the failure to release dopamine in the lateral olivocochlear efferent fibers may affect the synapse between inner hair cells and afferent dendrites of ganglion cells, thus altering the sound information conducted to the cochlear nucleus, 10 which can mainly damage the basal region of the cochlea.

Also, a recent study evaluated a family of five G protein-coupled dopamine receptors (D1, D2, D3, D4, and D5) in mice and observed the expression of these receptors in various cochlear regions, except for D3, with D2 receptors being expressed at much higher levels compared with the others. Thus, it is possible that dopaminergic suppression causes cochlear nerve fibers to be damaged when exposed to moderate sound pressure levels. 29 This finding provides new insights into the physiological alterations present in PD, which may underlie the hearing alterations observed in these patients.

Previous studies have reported an asymmetry of auditory function in PD, with greater impairment being observed in the ear ipsilateral to the most affected side by motor symptoms. 25 Another study also observed greater left-sided hearing impairment correlated with motor system asymmetry in PD, and this decline in peripheral auditory function was associated with basal ganglia dopamine transporter availability. 26

Unfortunately, no information was collected on the side most affected in PD patients and this was a limitation of this study, which should be considered in future studies. Despite this, although a higher average hearing threshold was observed in the left ear, this difference was only statistically significant for the frequency of 1 kHz, and it was also a difference observed in both patients with and without PD. Therefore, this asymmetry cannot be explained by PD and should be better studied in future studies.

Considering that PD mainly affects the elderly population, it may be that the hearing loss observed in these patients is due to age. In the present study, there was a positive correlation between the increase in hearing thresholds with increasing age; however, it was observed that the slope of worsening was greater among patients with PD, which suggests that PD may further aggravate the deterioration in hearing sensitivity in the elderly. Furthermore, in this study, care was taken to match the age of the groups to minimize the effects of this variable. Thus, considering that the patients in both groups were the same age, it is apparent that the higher thresholds observed at high frequencies were probably due to the deleterious effects of PD on the auditory system.

Despite the difference observed in the PTA, it is believed that this factor did not influence the electrophysiological responses, since the click frequencies used to record the ABR comprise frequencies between 2 and 4 kHz; likewise, for the CAEP with tone burst, tones of 1 and 2 kHz were used, and for the speech stimulus, the spectrum comprised the main speech frequencies, between 0.5 and 4 kHz.

About ABR no difference was found between the groups in either absolute or interpeak latencies, which suggests that the neural conduction time was still intact in the study population, similar to the CG individuals.

These findings corroborate previous studies that also did not find abnormal ABR latencies in PD patients, suggesting that their auditory pathways in the brainstem were intact. 3 12 27

However, unlike the present study, some authors reported slow electrophysiological responses of the neural auditory pathways in the brainstem of PD patients in studies that researched ABR. 24 30 31 32 33 34 The specialized literature reports that PD patients have abnormal acoustic information processing in the brainstem, which can be visualized more frequently by the increase in absolute latencies of waves III and V or latencies in interpeak intervals I-III and I-V. Even though the literature usually describes an increase in these interpeak latencies, the increase in interpeak interval I-III described in some studies cannot be dismissed. It suggested that PD patients may have changes in the auditory pathway in both the high and low brainstem. 24 30 31 32

The specialized literature reports that PD incidence and prevalence increase progressively after 60 years old. 23 In the present study, more than half of the patients were under 60 years old, as their median age was 58 years. This occurred because younger and less impaired patients had easier mobility and therefore were more willing to attend the place of examinations to participate in the research – which was a limitation of this study. This factor may explain the lack of abnormalities in the study population. Hence, further studies should be developed, assessing subjects older than 60 years.

Furthermore, according to Ferraz, 22 the use of levodopa is one of the most effective and viable ways of restoring neurotransmission, since levodopa penetrates the central nervous system and, through the action of the dopa decarboxylase enzyme, is converted into dopamine, helping with motor symptoms. Most articles on PD patients do not provide information on medication, which makes this analysis difficult. In this study, all the patients started treatment with Prolopa as soon as the diagnosis was confirmed. It is therefore believed that the use of the drug from the beginning of the disease may have slowed down the progression of the disease.

Similarly, the analysis of cortical processing speed showed no significant differences between the groups. This result corroborates another study, 3 which assessed the CAEP P3 component in patients with a previous diagnosis of PD, not finding latencies different from the normal limits for age. These authors also found intact auditory pathways in PD patients.

PD patients had smaller amplitudes than individuals without PD. P1-N1 amplitude was smaller with speech stimuli; P2-N2 amplitude was smaller with tone burst stimuli; and N2-P3 amplitude was smaller with both stimuli.

These results corroborate other studies in the literature, which also demonstrated decreased CAEP component amplitudes, particularly P3 35 36 37 38 and P2 amplitudes, 35 justifying that PD, its duration and severity, and attentional and cognitive disorders can impair the processing/habituation of new acoustic stimuli. This would decrease the number of responsive neurons and, consequently, wave amplitudes.

Although a previous study reported that PD patients had improved CAEP component amplitudes, especially P3 (increased amplitude), after drug treatment with dopamine, 39 the present study did not find the same result, as PD patients, even with drug therapy, still had smaller P2-N2 and N2-P3 amplitudes than individuals without PD. This corroborates the previous report 35 that did not demonstrate improved P2 and P3 amplitudes with the medicines.

It is known that the amplitude of the electrophysiological response is related to the number of neural fibers responsive to the stimulus. Thus, the present result suggests a decrease in neurons for cortical processing of acoustic information in PD, regardless of how long the disease has been present, the length of treatment, or the age of the patient.

According to Galhardo et al., 40 a review of the literature found that alterations in cognitive functions are present in PD, some of which are significantly reflected in language. Studies linking cognitive functions and PD have shown alterations in memory, language, visual-spatial ability, and executive functions, and have characterized PD as dementia, which often manifests its symptoms several years after the patient is diagnosed.

Studies with larger samples are needed, with a control group matched by gender and age, and which can also carry out longitudinal monitoring of the peripheral and central auditory pathways, correlating this with an assessment of the patient's clinical condition, with different dosages of medication and with the side that presented worse motor symptoms, since the absence of this data was a limitation of the present study.

It is known that CAEP is widely used and studied in various population profiles to investigate possible alterations in the processing of auditory information, especially in individuals with difficulties in behavioral tests due to various physical and cognitive impairments. Although the recording of CAEP is of the utmost importance, the literature consulted found few studies mentioning this assessment in individuals with PD.

Furthermore, the importance of otorhinolaryngological and speech and hearing assessment of these patients is highlighted to monitor the audiological responses of this population. Considering the results of this study together with those reported in the literature, the importance of assessing hearing thresholds using PTA is highlighted. This can be complemented by electrophysiological assessment using CAEP, which can help monitor the intervention and the evolution of the clinical condition.

Such assessments are often neglected, as other signs and symptoms are more prominent and become the main focus of attention for family members and professionals. However, considering the higher incidence of hearing loss in PD patients and the decline in peripheral hearing function, especially at higher frequencies, and central auditory skills, it is important to advise patients and their families about the difficulties of communicating due to hearing loss, as well as to advise them about strategies to facilitate communication, since they have altered acoustic processing of information. It is also worth highlighting the need for patients with hearing loss to be referred for rehabilitation fitting assistive listening devices since the brainstem neural response did not indicate any additional impairment for PD itself, which suggests a prognosis of success in the rehabilitation process.

In conclusion, the results of this study showed a significantly higher threshold in higher frequencies in patients with PD compared with patients without PD. The auditory evoked responses showed no differences between the groups at the brainstem level, however, the decrease in amplitude of all components in patients with PD in the CAEP, suggests a deficit in both automatic and attentional cortical processing of acoustic stimuli.

## References

[1] Schramm J MA Oliveira A F Leite I C Epidemiological transition and the study of burden of disease in Brazil Cien Saude Colet 2004 9 04 897 908

[2] de Lau L M Breteler M M Epidemiology of Parkinson's disease Lancet Neurol 2006 5 06 525 535 10.1016/S1474-4422(06)70471-9 16713924

[3] Pineroli J CA Campos D S Wiemes G R Meneses M S Mocellin M Auditory central avaliation in Parkinson Disease with BERA and P300 Braz J Otorhinolaryngol 2002 68 04 462 466 10.1590/S0034-72992002000400003

[4] Parkinson J An essay on the shaking palsy. 1817 J Neuropsychiatry Clin Neurosci 2002 14 02 223 236, discussion 222. 10.1176/jnp.14.2.223 11983801

[5] Dias A E Limongi J CP Tratamento dos distúrbios da voz na doença de Parkinson: o método Lee Silverman Arq Neuropsiquiatr 2003 61 01 61 66 10.1590/S0004-282X2003000100011 12715021

[6] Poewe W Non-motor symptoms in Parkinson's disease Eur J Neurol 2008 15(1, Suppl 1)14 20 10.1111/j.1468-1331.2008.02056.x 18353132

[7] Chaudhuri K R Schapira A HV Non-motor symptoms of Parkinson's disease: dopaminergic pathophysiology and treatment Lancet Neurol 2009 8 05 464 474 10.1016/S1474-4422(09)70068-7 19375664

[8] Krishnan S Sarma G Sarma S Kishore A Do nonmotor symptoms in Parkinson's disease differ from normal aging? Mov Disord 2011 26 11 2110 2113 10.1002/mds.23826 21661056

[9] Braak H Ghebremedhin E Rüb U Bratzke H Del Tredici K Stages in the development of Parkinson's disease-related pathology Cell Tissue Res 2004 318 01 121 134 10.1007/s00441-004-0956-9 15338272

[10] Pisani V Sisto R Moleti A An investigation of hearing impairment in de-novo Parkinson's disease patients: A preliminary study Parkinsonism Relat Disord 2015 21 08 987 991 10.1016/j.parkreldis.2015.06.007 26071125

[11] Livingston G Huntley J Sommerlad A Dementia prevention, intervention, and care: 2020 report of the Lancet Commission Lancet 2020 396(10248):413 446 10.1016/S0140-6736(20)30367-6Erratum in: Lancet. 2023 Sep 30;402(10408):1132. doi: 10.1016/S0140-6736(23)02043-3 32738937 PMC7392084

[12] Vitale C Marcelli V Allocca R Hearing impairment in Parkinson's disease: expanding the nonmotor phenotype Mov Disord 2012 27 12 1530 1535 10.1002/mds.25149 23032708

[13] Rabelo M B Lopes M S Corona A P Araújo R PC Alterações auditivas em indivíduos com doença de Parkinson Rev Ciênc Méd Biol 2014 13 03 319 324

[14] Vitale C Marcelli V Abate T Speech discrimination is impaired in parkinsonian patients: Expanding the audiologic findings of Parkinson's disease Parkinsonism Relat Disord 2016 22(1, Suppl 1)S138 S143 10.1016/j.parkreldis.2015.09.040 26421391

[15] Leme M S Função coclear na doença de Parkinson. Dissertação (Mestrado em Ciências da Reabilitação) - Faculdade de Medicina Universidade de São Paulo São Paulo 2021

[16] Jones C RG Malone T JL Dirnberger G Edwards M Jahanshahi M Basal ganglia, dopamine and temporal processing: performance on three timing tasks on and off medication in Parkinson's disease Brain Cogn 2008 68 01 30 41 10.1016/j.parkreldis.2015.09.040 18378374

[17] Geiser E Kaelin-Lang A The function of dopaminergic neural signal transmission in auditory pulse perception: evidence from dopaminergic treatment in Parkinson's patients Behav Brain Res 2011 225 01 270 275 10.1016/j.bbr.2011.07.019 21787806

[18] Hall J New handbook of auditory evoked responses Boston Allyn & Bacon 2006

[19] Lopes M S Rabelo M Corona A P Nobrega A C Araújo R PC Potenciais evocados auditivos do tronco encefálico na doença de Parkinson: revisão sistemática Rev Ciênc Méd Biol 2013 12 04 492 500 10.9771/cmbio.v12i4.9199

[20] Jerger J Clinical experience with impedance audiometry Arch Otolaryngol 1970 92 04 311 324 10.1001/archotol.1970.04310040005002 5455571

[21] Silman S Silverman C A Basic Audiológic Testing San Diego Singular Publishing Group 1997 44 52; 1997.

[22] Ferraz H B Tratamento da Doença de Parkinson Rev Neurocienc 1999 7 01 6 12 10.1590/S0004-282X1995000100001

[23] Clementino A CCR Ferreira N CP Borges N MS Epidemiological profile of people with Parkinson's disease BJS 2021 7 12 115963 115975 10.34117/bjdv7n12-384

[24] Yýlmaz S Karalý E Tokmak A Güçlü E Koçer A Oztürk O Auditory evaluation in Parkinsonian patients Eur Arch Otorhinolaryngol 2009 266 05 669 671 10.1007/s00405-009-0933-8 19263069

[25] Sisto R Viziano A Stefani A Lateralization of cochlear dysfunction as a specific biomarker of Parkinson's disease Brain Commun 2020 2 02 fcaa144 10.1093/braincomms/fcaa144 33376982 PMC7751021

[26] Garasto E Stefani A Pierantozzi M Association between hearing sensitivity and dopamine transporter availability in Parkinson's disease Brain Commun 2023 5 02 fcad075 10.1093/braincomms/fcad075 37006327 PMC10065189

[27] Shetty K Krishnan S Thulaseedharan J V Mohan M Kishore A Asymptomatic Hearing Impairment Frequently Occurs in Early-Onset Parkinson's Disease J Mov Disord 2019 12 02 84 90 10.14802/jmd.18048 30944288 PMC6547043

[28] Scarpa A Cassandro C Vitale C A comparison of auditory and vestibular dysfunction in Parkinson's disease and Multiple System Atrophy Parkinsonism Relat Disord 2020 71 51 57 10.1016/j.parkreldis.2020.01.018 32032926

[29] Maison S F Liu X P Eatock R A Sibley D R Grandy D K Liberman M C Dopaminergic signaling in the cochlea: receptor expression patterns and deletion phenotypes J Neurosci 2012 32 01 344 355 10.1523/JNEUROSCI.4720-11.2012 22219295 PMC3313790

[30] Podoshin L Ben-David J Fradis M Pratt H Brainstem auditory evoked potentials with and without increased stimulus rate as diagnostic tool in brainstem minor transient changes ORL J Otorhinolaryngol Relat Spec 1987 49 06 287 293 10.1159/000275954 3431838

[31] Fradis M Samet A Ben-David J Brainstem auditory evoked potentials to different stimulus rates in parkinsonian patients Eur Neurol 1988 28 04 181 186 10.1159/000116262 3416884

[32] Tachibana H Takeda M Sugita M Short-latency somatosensory and brainstem auditory evoked potentials in patients with Parkinson's disease Int J Neurosci 1989 44(3-4):321 326 10.3109/00207458908986210 2722418

[33] Liu C Zhang Y Tang W Wang B Wang B He S Evoked potential changes in patients with Parkinson's disease Brain Behav 2017 7 05 e00703 10.1002/brb3.703 28523237 PMC5434200

[34] Shalash A S Hassan D M Elrassas H H Auditory-and Vestibular-Evoked Potencials Correlate with Motor and Nom-Motor Features of Parkinson's Disease Front Neurol 2017 8 55 10.3389/fneur.2017.00055 28289399 PMC5326766

[35] Schomaker J Berendse H W Foncke E M Novelty processing and memory formation in Parkinson's disease Neuropsychologia 2014 62 124 136 10.1016/j.neuropsychologia.2014.07.016 25065495

[36] Solís-Vivanco R Rodríguez-Violante M Rodríguez-Agudelo Y Schilmann A Rodríguez-Ortiz U Ricardo-Garcell J The P3a wave: A reliable neurophysiological measure of Parkinson's disease duration and severity Clin Neurophysiol 2015 126 11 2142 2149 10.1016/j.clinph.2014.12.024 25655938

[37] Pauletti C Mannarelli D Locuratolo N Currà A Marinelli L Fattapposta F Central fatigue and attentional processing in Parkinson's disease: An event-related potentials study Clin Neurophysiol 2019 130 05 692 700 10.1016/j.clinph.2019.01.017 30875536

[38] Jafari Z Kolb B E Mohajerani M H Auditory Dysfunction in Parkinson's Disease Mov Disord 2020 35 04 537 550 10.1002/mds.28000 32052894

[39] Georgiev D Jahanshahi M Dreo J Čuš A Pirtošek Z Repovš G Dopaminergic medication alters auditory distractor processing in Parkinson's disease Acta Psychol (Amst) 2015 156 45 56 10.1016/j.actpsy.2015.02.001 25697781

[40] Galhardo M MAMC Amaral A KFJ Vieira A CC Characterizing cognitive disorders in Parkinson's disease Rev CEFAC 2009 11 02 251 257 10.1590/S1516-18462009000600015

